# Trend, population structure, and trait mapping from 15 years of national varietal trials of UK winter wheat

**DOI:** 10.1093/g3journal/jkab415

**Published:** 2021-12-13

**Authors:** Oluwaseyi Shorinola, James Simmonds, Luzie U Wingen, Cristobal Uauy

**Affiliations:** 1 Crop Genetics Department, John Innes Centre, Norwich Research Park, Norwich NR4 7UH, UK; 2 Bioscience Eastern and Central Africa—International Livestock Research Institute (BecA-ILRI), Nairobi 00100, Kenya

**Keywords:** wheat, SNP, GWAS, *NAM-A1*, trend analysis

## Abstract

There are now a rich variety of genomic and genotypic resources available to wheat researchers and breeders. However, the generation of high-quality and field-relevant phenotyping data which is required to capture the complexities of gene × environment interactions remains a major bottleneck. Historical datasets from national variety performance trials (NVPT) provide sufficient dimensions, in terms of numbers of years and locations, to examine phenotypic trends and study gene × environment interactions. Using NVPT for winter wheat varieties grown in the United Kingdom between 2002 and 2017, we examined temporal trends for eight traits related to yield, adaptation, and grain quality performance. We show a non-stationary linear trend for yield, grain protein content, Hagberg Falling Number (HFN), and days to ripening. Our data also show high environmental stability for yield, grain protein content, and specific weight in UK winter wheat varieties and high environmental sensitivity for HFN. We also show that UK varieties released within this period cluster into four main population groups. Using the historical NVPT data in a genome-wide association analysis, we uncovered a significant marker-trait association peak on wheat chromosome 6A spanning the *NAM-A1* gene that have been previously associated with early senescence. Together, our results show the value of utilizing the data routinely collected during national variety evaluation process for examining breeding progress and the genetic architecture of important traits.

## Introduction 

Over the last 3 years, there has been a rapid surge in the development of genomic resources for wheat (reviewed in Adamski *et al.* 2020). This includes a chromosome-scale reference assembly of the Chinese Spring cultivar (RefSeqv1) and a pan-genome resource comprised of chromosome and scaffold-level assemblies of 15 hexaploid wheat cultivars [International Wheat Genome Sequencing Consortium (IWGSC) *et al.* 2018; Walkowiak *et al.* 2020]. There is also a wide range of array-based (Axiom-35K, iSelect 90K, Axiom-660K, and Axiom-820K; Wang *et al.* 2014; Winfield *et al.* 2016; Allen *et al.* 2017), sequencing-based (*e.g.*, DARTSeq, RADSeq) or PCR-based (*e.g.*, KASP, TaqMan, rhAmp; Semagn *et al.* 2014; Ayalew *et al.* 2019) SNP genotyping assays available to wheat researchers and breeders. There have also been efforts to resequence different wheat populations either through reduced-representation sequencing approach like exome-capture and sequencing (*e.g.*, Jordan *et al.* 2015; Krasileva *et al.* 2017; He *et al.* 2019) or through whole-genome resequencing (*e.g.*, Cheng *et al.* 2019; Scott *et al.* 2021). This preponderance of genomics and genotypic data, which are available in open-access repositories (*e.g.*, EnsemblPlants, CerealsDB; Bolser *et al.* 2016; Howe *et al.* 2020; Wilkinson *et al.* 2020), now makes it possible to map traits at high-resolution (*e.g.*, Walkowiak *et al.* 2020), examine population diversity at whole-genome levels or in breeding units (haplotypes: *e.g.*, Brinton *et al.* 2020; Scott *et al.* 2021), and implement genome-assisted breeding schemes using marker-assisted and/or genomic selection (*e.g.*, Rasheed and Xia 2019; Sweeney *et al.* 2019).

Despite these advances, the generation of high-quality and field-relevant phenotyping data remains a major bottleneck. Modern phenomics platforms have improved phenotyping throughput and precision under controlled conditions, but these do not always capture the environmental effects experienced under real-world farming conditions (Yang *et al.* 2020). Given climate change projections of fluctuating radiation, heat, and precipitation patterns in major wheat-growing areas (including the United Kingdom; Semenov 2009; Trnka *et al.* 2019), breeding for phenotypic stability and understanding complex gene × environment interactions is of high priority.

Due to their large scale and multi-environment (years and locations) design, historical dataset from national variety performance trials (NVPT) provides sufficient dimensions, in terms of years and locations to examine phenotypic trends and study gene × environment interactions. These historical datasets are, however, incomplete by design because of, for example, changes in the number and specific set of varieties trialed and changes in the field sites used from year to year. Previous studies have analyzed NPVT data for wheat and barley in the United Kingdom (Silvey 1981; Mackay *et al.* 2011; Looseley *et al.* 2020) and similar analyses of historical data have been conducted elsewhere (*e.g.*, Crossa *et al.* 2007; Pozniak *et al.* 2012; Laidig *et al.* 2021).

In the United Kingdom, new wheat varieties undergo statutory tests before they are registered on the National List (NL). Registered varieties are subsequently introduced (or maintained on) the UK Recommended List (RL) after undergoing independent non-statutory NPVT managed by the Agriculture and Horticulture Development Board (AHDB, formerly Home-Grown Cereals Authority). The NL serves as variety registry while the RL is used as a reference by farmers for variety selection. Mackay *et al.* (2011) reanalyzed data from the UK NL and RL trials conducted between 1948 and 2007, and found significant yield improvement that was mostly attributed to plant breeding. In this study, we analyzed data from the UK RL NVPT for winter wheat between 2002 and 2017 and used this to examine temporal trends in eight yield, adaptation, and grain quality traits. We also demonstrate the usefulness of these NVPT dataset for trait mapping to uncover loci of breeding importance.

## Materials and methods

### NVPT datasets

We downloaded result files for the NVPT for winter wheat in the United Kingdom from 2002 to 2010 and 2012 to 2017 from the AHDB website containing harvest result for the RLs for cereals and oilseeds (accessible at: https://ahdb.org.uk/knowledge-library/recommended-lists-for-cereals-and-oilseeds-rl-harvest-results-archive; Accessed: 2021 December 13). We focused our study on data for eight traits including yield, adaptation and grain quality traits. Yield and height data were collected from treated and untreated trials. The treated trials included management for diseases (fungicide spray) while the untreated trials did not include disease management. Both trials were managed under standard husbandry practices including the application of plant growth regulator (PGR), herbicide, fertilizer, and pest control management as recommended by AHDB. Details of the AHDB RL trial protocol is accessible at: https://ahdb.org.uk/ahdb-recommended-lists-for-cereals-and-oilseeds-2016-2021 (Accessed: 2021 December 13). Before analyses, we filtered the dataset to remove observations with unknown locations or from locations where trials were abandoned. Varieties that were trialed in a single year were also omitted. The nomenclature of varieties, locations, and counties were standardized in cases where different designation or acronyms were used for the same variety, location or county across different years. After filtering, the distribution of the observations obtained for each of the eight target traits resembles a bell curve suggesting normal distribution (Supplementary Figure S1).

### Germplasm

Data for a total of 168 varieties were used in this study. These include 133 varieties whose phenotype information was obtained from the AHDB website as described above. An additional 35 winter wheat varieties released in the United Kingdom before 2002 were included for genotyping using the Axiom-35K array as described below. The number of varieties used for each analysis in this study is detailed in Supplementary Figure S2.

### Statistical analyses

We used a two-stage approach to examine the linear trend of traits from the NVPT data. First, we fitted a linear mixed model (LMM) to the NVPT data using restricted maximum likelihood estimation. The model was implemented using the lme4 package in *R* as:

$$Y_{\mathrm{ijk}}=µ+v_{i}+y_{j}+s_{jk}+e_{\mathrm{ijk}}.$$

*Y_ijk_* is the historical performance of variety *i* in year *j* at location *k*. *µ* is the overall mean performance of all varieties, *v_i_* is the effect of variety *i*, *y_j_* is the effect of year *j* (the calendar year of the trial harvest) and *S_jk_* is the effect of location *k* within year *j*. *e_ijk_* is the residual variance arising from factors not accounted for in the model including variety × year interaction. As our main interest was the performance for each variety, the variety effect was fitted as fixed factor while the year and site (nested within year) were fitted as random factors. This is slightly different to the strategy used by Mackay *et al.* (2011), which also included calendar year as a fixed factor to account for the long year interval (1948–2002) examined and changes in trial management system across these years. We derived estimates for the varieties means (hereafter referred to as EVM) from the LMM. Second, we used a linear model to regress the EVM derived from the LMM above against the year the variety was first entered into the NVPT.

For trait comparison between end-use groups, analysis of variance followed by *post* *hoc* TukeyHSD was used to evaluate and compare significant difference in EVM of varieties belonging to different end-use groups. The lstrend function implemented in the *R* lsmeans package (Lenth 2016) was used to estimate and compare slopes of the linear regression between groups. For slope comparisons between the four end-use groups, the adjusted *P*-value is presented based on Tukey’s method of comparison.

We used the Finlay Wilkinson (FW) regression to examine phenotype stability (Finlay and Wilkinson 1963). The original FW regression is not best suited for data from incomplete trial design as the environment means used for normalizing varietal performance are biased due to incomplete replication of varieties across all environments. To circumvent this bias in our analysis, we used the Bayesian method proposed by Su *et al.* (2006) and implemented in the *R* package FW (Lian and de los Campos 2015). Only varieties that were trialed in three or more years were used for this analysis. The mean values for each variety in each year were used as input. The model was fitted with the Bayesian “gibbs” method, with 50,000 iterations and 5000 burnIn rate as suggested for wheat analyses in the FW package paper (Lian and de los Campos 2015). The FW coefficients are presented as b + 1 which describes expected change in variety performance per unit change of the environment effect (Lian and de los Campos 2015).

### Genotyping, population structure, and association analysis

A total of 139 varieties (104 varieties from the NVPT and 35 pre-2002 varieties) were genotyped using the Axiom-35K array (Allen *et al.* 2017). We filtered the genotype data to include only sites with >0.1 minor allele frequencies and no heterozygous calls (as most loci are expected to be fixed in pure lines). The markers were also filtered to remove loci with high linkage disequilibrium (*R*^2^ > 0.8) within each 20-SNP window using PLINK 1.9 (Chang *et al.* 2015). This was done to remove biases arising from high LD linkage blocks containing large numbers of markers (such as from introgression from wild relatives). These high LD linkage blocks can bias the contributions of such loci in population structure analysis.

To assign physical positions to the Axiom markers, their sequences were used as queries in BLASTn alignments against the IWGSC RefSeqv1.0 assembly [International Wheat Genome Sequencing Consortium (IWGSC) *et al.* 2018] as described in Brinton *et al.* (2020) and the best hits on each of the three wheat homoeologous genomes (A, B, and D) were recorded. Of these, the correct homoeologous chromosome was selected using genetic mapping information from 13 populations (Gardiner *et al.* 2019) where available for each marker. Otherwise, the highest BLASTn score was used to select the homoeologous chromosome. In case of conflicting genetic mapping results for the correct chromosome between the mapping populations, the most frequent outcome was used.

Population structure analysis was done using discriminant analysis of principal component (DAPC) as implemented in the Adegenet *R* package (Jombart and Ahmed 2011). For this, the number of population cluster (*k*) was determined by kmeans clustering using a range of *k*. The *k* with the minimum Bayesian Information Criterion and that minimizes overlap between groups was selected as the optimum *k*. To increase the accuracy of grouping, 50 iterations of the kmeans clustering algorithm were run and the population group to which a variety was most frequently assigned was selected. Also, the cross-validation function (xvalDapc) was used to select the optimum number of principal components to use for DAPC.

We used a K+Q LMM-based model for genome-wide association analysis (GWAs) using the GAPIT3 *R* package (Wang and Zhang 2021). K represents the kinship matrix describing the relatedness between the varieties and Q represents the population grouping derived from the DAPC analysis described above. A false discovery rate (FDR) threshold with adjusted *P*-value below 0.05 was used to select markers with significant association with the trait of interest.

## Results

### Estimates from multi-environment trials capture expected relationship between traits

**Figure 1:**
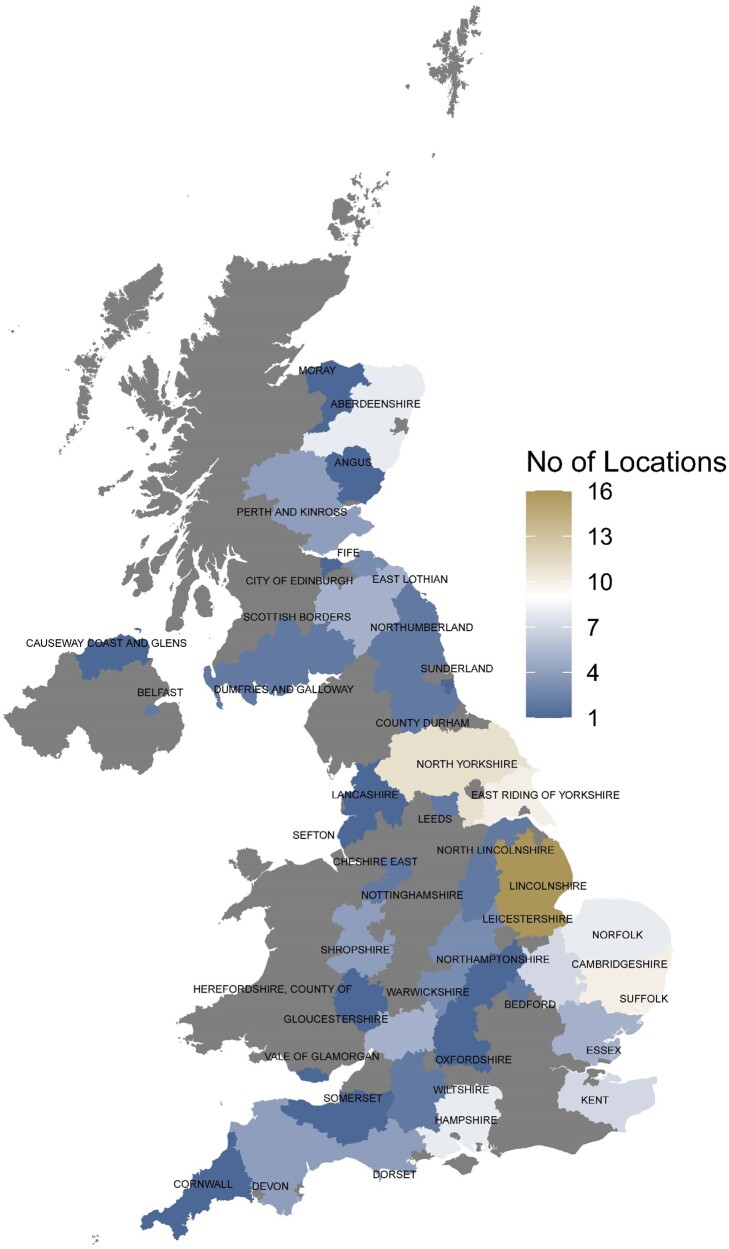
Distribution of 162 NVPT locations used in this study (2002 and 2017). The number of field sites within each county and unitary authority is indicated in color.

We analyzed historical data set of the UK RL NVPT from 2002 to 2017. We focused our analyses on six traits of agronomic and economic importance: yield, plant height, days to ripening, Hagberg falling number (HFN; Krupnov and Krupnova 2016), grain protein content and specific weight. For yield and plant height, we analyzed data coming from (fungicide) treated and untreated trials. This results in a final dataset for eight traits. After quality controls (described in *Materials and Methods*), we retained 52,152 observations for these eight traits from 133 winter wheat varieties (Supplementary Table S1). These 133 varieties were phenotyped in at least two years across a combined 162 locations, with a subset of 95 locations being used for evaluations in two or more years. Table 1 details the number of varieties phenotyped for each trait and the number of locations and year-location combinations used. The trial locations were spread across 43 counties and unitary authorities in England, Wales, Scotland, and Northern Ireland as shown in Figure 1.

**Table 1:** Number of varieties, sites, and years of trials for the UK NVPT between 2002 and 2017

Trait	No. of varieties^*a*^	Trial years per trait	Mean trial year per variety	No. of trial locations^*b*^	Year × location combinations	Total observations
Treated yield	133	15	4	158	410	13,080
Untreated yield	131	15	4	53	124	4,156
Protein content	128	15	4	99	230	7,142
Days to ripening	133	15	4	108	247	7,977
HFN	128	15	4	99	227	7,154
Specific weight	129	15	4	99	231	7,091
Treated height	108	11	4	74	171	3,905
Untreated height	107	11	4	30	75	1,647

a Not all varieties were tested for each trait, and in each year and location.

b Some locations were used in more than one year.

**Figure 2:**
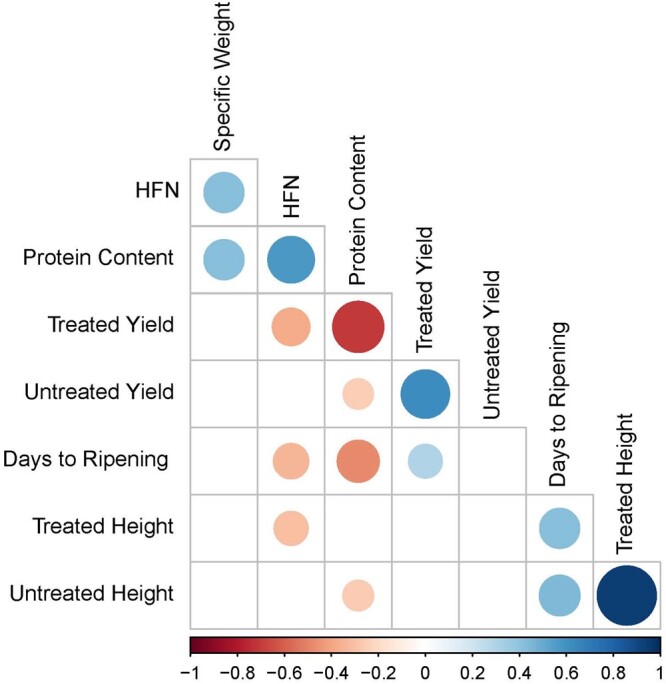
Phenotype correlation between yield, adaptation, and grain quality traits. EVM were derived for each variety from the NVPT conducted between 2002 and 2017. Only significant correlations (*P* < 0.05) are indicated. Positive and negative correlations are indicated with the blue and red circles, respectively, with the size and color intensity of the circles representing the magnitude of the correlation.

Using a LMM that accounted for variation arising from the different years and locations of the trials, we derived EVM for each variety for each trait (Supplementary Table S2). Correlation analysis using the EVM captured expected patterns of relationship between the measured traits (Figure 2). We observed significant positive correlations between treated and untreated trials for height and yield, although the correlation between treated/untreated trials for height (0.94) was much stronger than for yield (0.63). HFN and grain protein content were positively correlated to each other, but negatively correlated to treated yield, treated plant height and days to ripening.

### Examining trait trends

**Figure 3:**
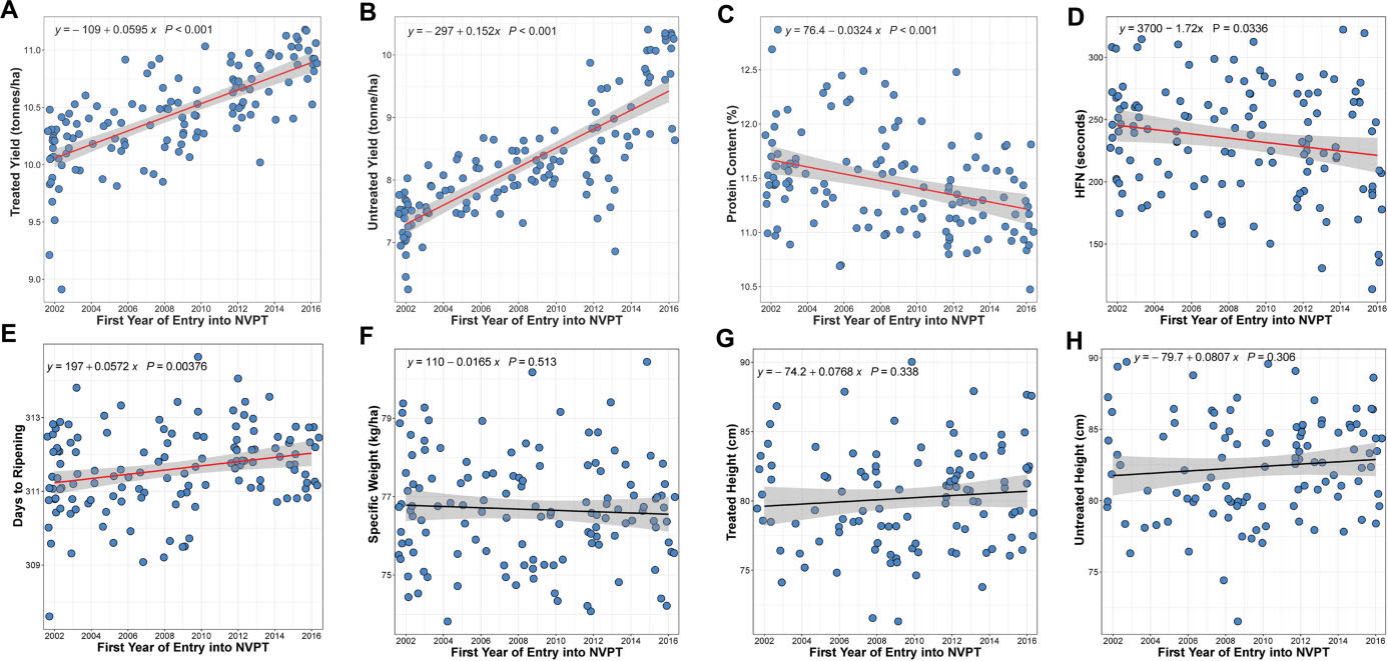
Temporal trait trend in UK winter wheat. Scatter plot showing changes in yield in the treated (A) and untreated trial (B), protein content (C), HFN (D), days to ripening (E), specific weight (F), plant height in treated (G) and untreated (H) trials. Each blue dots represent an individual variety. For each trait, the EVM for each variety is regressed against the first year of entry in the 2002–2017 trials. The solid line shows the regression line of the linear model and is colored red if significant (*P* < 0.05). The shaded region defines the confidence interval. The regression equation is shown within each plot. The EVM data used for these plots are in Supplementary Table S2.

We next examined the temporal pattern across the 15 years of trials to highlight linear trends in traits due to breeding progress. For this, we regressed the EVM for each variety on its year of first entry to the NVPT which is directly related to its year of release. This regression likely captures temporal pattern of breeding progress as successive releases of varieties are expected to outperform previous releases in one or more traits. We observed linear increase for yield between 2002 and 2017 in both the treated and untreated trials (Figure 3, A and B). The rate of yield increase in the untreated trial was significantly higher than in the treated trials (rate difference = 0.093 tonnes/ha/year, *P* < 0.0001). Conversely, grain protein content and HFN showed small but significant decrease over time (*P* < 0.001 and 0.03, respectively; Figure 3, C and D). We also observed a significant delay in days to ripening over the same period (*P* = 0.004, Figure 3E). Changes in plant height (treated and untreated) and specific weight were not significant (*P* = 0.31–0.51, Figure 3, F–H) suggesting stable trends.

**Figure 4:**
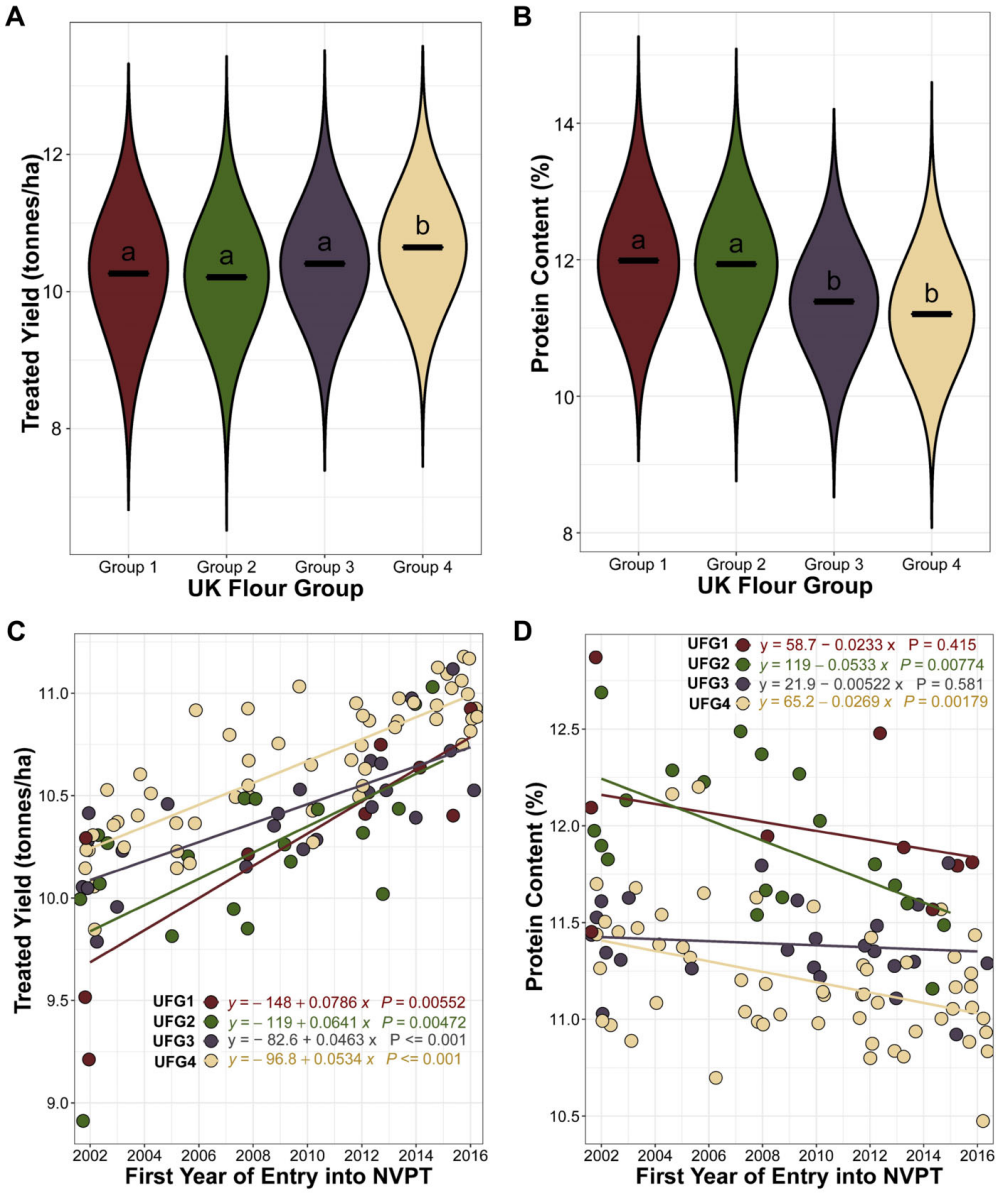
Temporal trait trend by end-use groups. (A, B) Violin plots showing distribution for yield (A) and protein content (B) for the different end-use groups. The solid lines represent the mean of the distribution and the black letters show Tukey statistical comparison between the groups. Groups that are statistically similar share the same letter. (C, D) Scatter plot showing changes in yield (C) and protein content (D) for each end-use group of UK winter wheat. Each dot represents a variety while the colors of the dots represent the end-use groups (UK Flour Groups 1–4). For each trait, the EVM for each variety is regressed against the first year of entry in the 2002–2017 trials. The solid lines are the regression line of the linear model. The regression line equation for each group is shown. UFG1, UFG2, UFG3, and UFG4 are represented by the red, green, gray and peach dots, lines and text, respectively.

UK wheat varieties are classified into four main end-use groups as described by the UK Flour Millers (www.ukflourmillers.org; Accessed: 2021 December 13). These include the UK Flour Groups 1–4, hereafter referred to as UFG1–4. The UFG1 and UFG2 varieties have superior grain quality (grain protein content and HFN) and are used for breadmaking. UFG3 varieties are often used for biscuits and cakes, whereas UFG4 varieties usually have high yield potential but inferior grain quality and are mainly used for animal feed. As yield and protein content are important measures for these end-use classifications, we examined how the temporal trends observed for these traits varied for the different end-use groups. Expectedly, UFG4 varieties showed higher yield while the breadmaking varieties (UFG1-2) show higher grain protein content (Figure 4, A and B). All end-use groups showed a significant increasing yield trend across time and the rates of increase were not significantly different between the end-use groups (*P* = 0.263–0.885; Figure 4C). UFG2 and UFG4 varieties showed a significant and comparable decline in grain protein content over time (Figure 4D) while changes in protein content of UFG1 and UFG3 varieties were non-significant (Figure 4D).

### Yield, protein content, specific weight, but not HFN, are stable in UK environments

**Figure 5:**
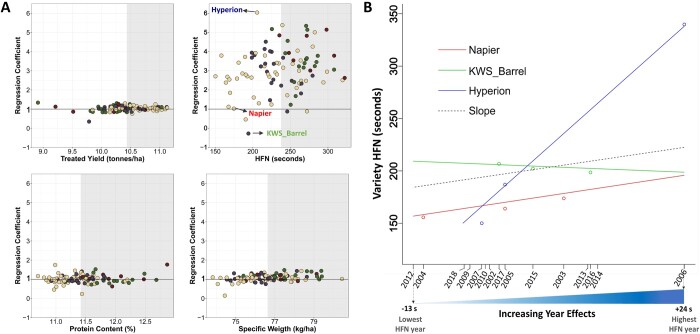
Phenotype stability by end-use group. Scatter plot showing stability for treated yield, protein content, specific weight and HFN for UK winter wheat varieties across the 15 years of trials (2002–2017). The *y*-axis represents the Finlay Wilkinson coefficient which specifies expected change in performance per unit change in environment (year) effect. Varieties with above median trait performance are in the shaded region. The solid line indicates stable performance in all environments *i.e.*, b + 1 = 1 (Lian and de los Campos 2015). Datapoints for three varieties whose HFN performance are further illustrated in panel B are labeled. (B) Plot of HFN performance of varieties with lowest, highest and stable (∼1) FW coefficient against the estimated environment year effect. The dashed lines present a constant slope of 1.

Using a modified FW regression (Lian and de los Campos 2015) for measuring genotype × environment interaction, we examined the stability of yield and end-use quality traits across the trial years (Figure 5, Supplementary Table S3). Only 95 varieties that were trialed in 3 or more years were included in this analysis. FW regression measures the stability of variety performance across different environments by regressing individual variety trait means on the environmental effect (Finlay and Wilkinson 1963). FW regression coefficient close to 1 suggests average varietal stability in which variety performance is consistent with environment effect *i.e.*, variety performs poorly in bad environments and well in good environments. Larger values suggest below average stability *i.e.*, higher environmental sensitivity.

Yield was stable across years in most UK wheat varieties (regression coefficients close to 1, Figure 5A). Similarly, most of the varieties examined showed high stability in protein content and specific weight, with bread-making varieties stably producing grains with above median protein and specific weights (Figure 5A). HFN, on the other hand, showed varying FW coefficients ranging from −0.28 to 6.03. More than 83% of the 95 UK wheat varieties examined have FW coefficient > 2 for HFN suggesting below-average stability. To illustrate this, Figure 5B shows the HFN performance of three varieties with different FW coefficients: KWS_Barrel, Hyperion, and Napier. Napier which has a FW coefficient of 1.02 consistently showed low HFN values in all the years it was trialed. On the other hand, Hyperion with the highest FW co-efficient (6.03), showed extreme HFN phenotypes. That is, very low HFN value in low-HFN years and very high-HFN value in high-HFN years suggesting high environmental sensitivity. KWS_Barrel’s with the lowest FW co-efficient (−0.28) showed HFN phenotypes that was fairly constant irrespective of the environments it was trialed.

### Post-2002 UK wheat varieties belong to four distinct population groups

**Figure 6:**
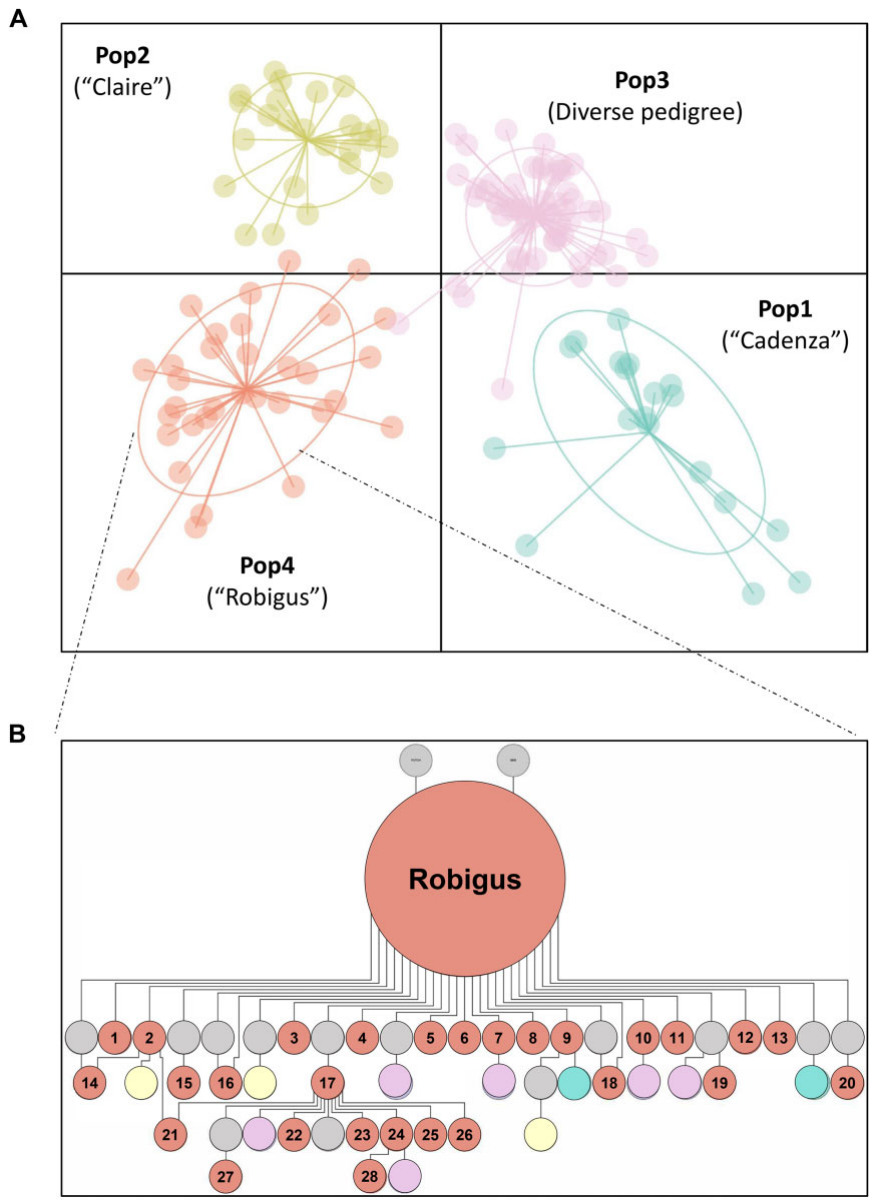
Population structure of UK winter wheat varieties using DAPC analysis. (A) The representative variety for each population group (Pop) is indicated except for Pop2 which consists of a more diverse pedigree. (B) Pedigree structure for Pop4 (“Robigus” group). The number in the inset represent varieties: (1) Invicta (2) Lear (3) Torch (4) Twister (5) Britannia (6) Gravitas (7) Conqueror (8) Cougar (9) Viscount (10) Qplus (11) Warrior (12) Zulu (13) Leeds (14) Panacea (15) Tuxedo (16) KWS_Target (17) Oakley (18) KWS_Croft (19) Evolution (20) Icon (21) Energise (22) Rgt_Scrummage (23) Horatio (24) KWS_Santiago (25) Reflection (26) KWS_Gator (27) KWS_Conversion (28) KWS_Kerrin. The population groups are represented by teal (Pop1), yellow (Pop2), purple (Pop3), and red (Pop4) circles, whereas gray circles represent varieties which were not genotyped in this study.

Using the Axiom35k SNP array (Allen *et al.* 2017), we genotyped 139 varieties including a subset of those trialed between 2002 and 2017 (104) and additional historic UK wheat cultivars. After quality filtering (described in *Materials and Methods*), we selected 4039 high-quality markers dataset (Supplementary Table S4) including 1623, 1841, and 589 markers on the A, B, and D sub-genomes, respectively (Supplementary Table S5). Using these genotypic data, we examined the population structure within the UK wheat collection. DAPC analysis revealed four distinct population groups (Pop1-4; Figure 6A, Supplementary Table S6). Using Helium for pedigree visualization (Shaw *et al.* 2014; Helium pedigree information provided as Supplementary file), we could trace important parents for three (Pop1, 2, and 4) of the four population groups. Pop1 contains 20 varieties, of which 16 (80%) have Cadenza in their pedigree. This is consistent with Cadenza being an important parent for Pop1. Pop2 comprises 26 varieties, 20 (77%) of which contain Claire in their pedigree. Pop4 includes 32 varieties, 29 (91%) of which trace their pedigree to Robigus, suggesting Robigus as an important parent for this group (Figure 6B). Pop3 is the largest group with 61 varieties. We could not identify a pre-dominant variety parent for this population group.

Using a subset of 111 varieties with both genotype and end-use group information (Supplementary Figure S2), we examined the association between the population groups and end-use groups (Supplementary Figure S3). The “Claire” (Pop2) and “Robigus” (Pop4) population groups contain mostly (98%) UFG3 and UFG4 varieties used for biscuit/cakes and feeds, respectively. While the “Cadenza” (Pop1) population group mostly (71%) contain UFG1 and UFG2 varieties used for breadmaking.

### Using NVPT data for trait mapping

**Figure 7:**
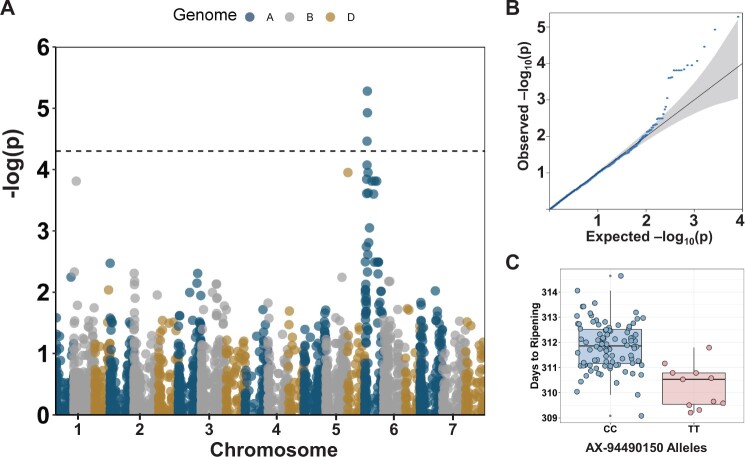
(A) Manhattan plot for days to ripening using EVM derived from the 2002–2017 NVPT of UK winter wheat varieties. The FDR threshold is indicated with a dotted line. The seven wheat chromosome groups are indicated on the *x*-axis and each homoeologous sub-genome is colored in red (A genome), gray (B), or yellow (D). (B) QQplot showing expected and observed distribution of –log (*P*-values). (C) Allele effect of the marker showing the highest significant MTA for days to ripening.

We next examined if the EVM obtained from the NVPT could be used for trait mapping through (GWAS). Only 104 varieties with both phenotype (from the 2002 to 2017 NVPT) and genotype data were used for GWAS. Our GWAS analysis identified a region on the short arm of chromosome 6A with significant marker-trait association (MTA) for days to ripening (Figure 7, A and B). The days to ripening MTA region contain three markers, AX-94913053, AX-94490150, and AX-94710688, located in an interval (73.6 – 86.5 Mbp) containing the *NAM-A1* gene (*TraesCS6A02G108300*; 77.1 Mbp) that is associated with variation in senescence in European wheat cultivars (Cormier *et al.* 2015). Days to ripening was significantly (P < 0.0001) different between the allele groups of AX-94490150 (83.6 Mbp) which has the highest significance score (Figure 7C). The 11 varieties containing the early maturity “T” alleles of AX-94490150 belong to the four different population groups previously defined. Interestingly, all 11 varieties descended from Moulin (released in 1985, Supplementary Figure S4) which also has the early maturity “T” allele (based on genotype data from CerealsDB; Allen *et al.* 2017).

## Discussions

### Yield is an important driver of linear trends

Using historical data from UK NVPT, we examined phenotypic trends in winter wheat varieties trialed between 2002 and 2017. Our analysis highlights a linear increase for yield (treated and untreated) and days to ripening, and a linear decrease in protein content and HFN. Given that the model used to analyze this data adjusted for variation arising from locations across years, and that agronomic practices are largely consistent in the NVPT, this linear trend can be attributed mostly to genetic improvement of varieties over time. Mackay *et al.* (2011) similarly attributed 88% of yield increase in cereals crops in the United Kingdom from 1982 to 2007 to genetic improvement. Yield is the most important determinant of grain market value; as such the linear increase in yield is consistent with concerted breeding efforts to improve yield under UK wheat growing conditions. In addition to the overall yield trend, we also observed consistent and similar linear increases in yield in all the four UK Flour Groups (UFG1–4). This further highlight yield as the main breeding target for varietal development (and adoption into the RL) irrespective of their target end-use groups.

We observed that the rate of yield increase in untreated trials (152 kg/ha/year) is significantly (*P* < 0.0001) higher than in treated trials (59.5 kg/ha/year) across the 15-year period. Mackay *et al.* (2011) similarly observed the same pattern between 1982 and 2007 and argued that this pattern is due to loss of disease resistance by some varieties during the trial period examined. Varieties typically progressively lose resistance over time (Meikle and Scarisbrick 1994) and consequently variety performance declines with time. This means that under untreated trial conditions, newly introduced varieties with “intact” disease resistance will outperform a portion of previously released varieties whose disease resistance have “broken down.” This differential loss of disease resistance will further increase the variation in variety yield performance in untreated trials in addition to the variation arising from non disease-related genetic factors observed in treated trials. In other words, there is an “upward bias” in variety effects for the yield observed in untreated trials as described by Mackay *et al.* (2011).

**Figure 8:**
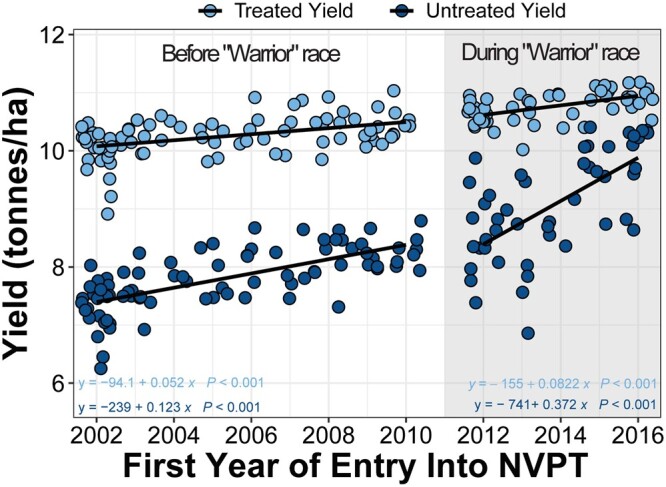
Yield comparison between treated and untreated trials before and after the emergence of the “warrior” yellow rust race. Scatter plot showing changes in yield in treated (light blue) and untreated trials (dark blue) before (unshaded region) and after (shaded region) the emergence of the “warrior” yellow rust race. The EVM for each period are regressed separately against the first year of entry into the NVPT trials for each variety. The solid lines are the regression lines. The regression equations are shown at the bottom corners of the plot.

Based on the rationale described above, it would be expected that a sudden loss of resistance in a large proportion of varieties due to the emergence of a more virulent pathogen race would result in a marked upward bias in variety effect estimates. This is what we observed when we compared yield trends before and after the emergence of the yellow rust (*Puccinia striiformis*) “Warrior” race in 2011 (Hubbard *et al.* 2015). The rate of yield increase in untreated trials significantly (*P* < 0.001) increased threefold from 123 kg/ha/year before the emergence of the “Warrior” race to 372 kg/ha/year after the emergence of the “Warrior” race (Figure 8). During the same time, the rate of yield increase was significantly (*P* = 0.2697) comparable in the treated trial before and after the emergence of the “Warrior” race (Figure 8). The use of historical data in this study allowed us to identify this trend and thus highlight the importance of such datasets for dissecting the effect of important events in a national cropping history such as change in disease epidemics.

It is also interesting to speculate that the higher rate of yield increase observed in the untreated trials indirectly suggests that newer varieties contain new sources of genetic resistance that improve their performance over older varieties. This is likely not accidental, but points to concerted efforts by breeders to introduce more effective source of genetic resistance into UK wheat. The improved genetic resistance profile of newer varieties narrows the yield gap observed between the treated and untreated trials (Figure 8). We cannot, however, rule out the fact that this narrower yield gap might be due to lower disease pressure in recent years. A more detailed genetic characterization will be needed to accurately describe the genetic resistance profile of UK wheat varieties.

Concomitant with the yield increase, there has been a decrease in grain protein content from 2002 to 2017 which reflects the well-established antagonistic relationship between yield and protein content (Supplementary Figure S5; Simmonds 1995). Unlike for yield, linear trends in grain protein content were not consistent across the four end-use groups. While we identified an overall significant decrease in grain protein content over time, this was not observed in the UFG1 varieties that are used for breadmaking (Figure 4). UFG2 varieties which also have breadmaking potential, however, showed significant decrease over time just like the UFG4 varieties used for animal feed. The decline in UFG2 varieties grain protein content may be due to the fact that this group comprise varieties that did not consistently meet the higher grain quality (in particular protein content) requirement for UFG1 and were downgraded to UFG2. The fact that our analysis captures expected trait (yield, protein content, and HFN) differences in end-use groups (Figure 4, A and B, Supplementary Figure S6, A and B) suggests that the linear mixed effect model adopted is appropriate to handle the incomplete design of the NVPT and to examine phenotype trends within each end-use group.

### HFN stability is still an important breeding target

The multi-dimensional (year and location) nature of the NVPT also allows for examining varietal adaptability across multiple environments. Despite the unbalanced nature of the NVPT dataset, results from our yield stability analyses using this dataset largely agrees with findings by Pennacchi et al. (2019) who examined yield stability using a balanced dataset from three years of field trials on 64 UK wheat varieties that were released between 1975 and 2008. Pennacchi et al. (2019) identified four varieties (Gladiator, Humber, Mercato, and Zebedee) as high-yielding varieties with high stability. Three out of these four varieties are present in our study (Gladiator, Humber, and Zebedee), of which two (Humber and Zebedee) are among the top five varieties with highest yield stability. Similarly, Istabraq which was identified by Pennacchi et al. (2019) as less stable was one of the varieties with the least yield stability in our study.

Despite this variation in yield stability, we generally observed year-to-year stability in yield (0.35–1.34) and protein content (0.23–1.77) in most of the varieties irrespective of their end-use group. This is likely attributable to the fact that we mainly examined data from RL trials that are comprised of varieties which had been previously screened for distinctness, uniformity, and stability during National Listing trials. Despite this “pre-screening,” almost all the varieties show high environmental sensitivity for HFN (FW coefficient: −0.28 to 6.03). Sjoberg *et al.* (2020) similarly obtained a wide range of FW coefficient for HFN in 133 varieties trialed across 3 years in the Pacific Northwest of the United States.

HFN is inversely related to α-amylase activity within the grain. High α-amylase activity caused by incidences of pre-harvest sprouting (PHS) and/or pre-maturity amylase (PMA) reduce the bread-making potential of wheat grains. Both PHS and PMA are known to be highly environmental dependent: PHS is induced by wet raining conditions during harvest maturity while PMA is mostly caused by low or high-temperature shock around grain physiological maturity (Joe *et al.* 2005; Mares and Mrva 2014). The environmental conditions required to induce PHS and PMA occur infrequently from year to year making it difficult for breeders to screen for these traits under field conditions. In addition, both traits are controlled by many genes most of which have small effects making marker-assisted selection (MAS) for HFN stability difficult. Within the last decade, progress has been made in identifying genes with major effects on PHS including *TaMFT* and *TaMKK3*-A (Nakamura *et al.* 2011; Torada *et al.* 2016). We also previously showed the effect of *TaMMK3*-*A* in reducing PHS in UK germplasm (Shorinola *et al.* 2016) and developed markers to facilitates its use in breeding (Shorinola *et al.* 2017). The availability of markers for major genes controlling PHS now makes it possible to apply MAS for improving HFN. However, selection for PMA resistance remains a major challenge because the conditions that induce PMA vary between varieties (Liu *et al.* 2021).

### Population structure within UK winter wheat germplasm

Our analysis reveals that three modern wheat varieties made major contributions to the development of winter wheat varieties released in the United Kingdom between 2002 and 2017. These include Cadenza (Pop1), Claire (Pop2) and Robigus (Pop4), which are themselves UK varieties released in 1992, 1999, 2005, respectively. In fact, more than 75% of the variety parents (*i.e.*, released varieties used as direct parents in crosses) used to generate the UK RL varieties released between 2002 and 2017 are from the United Kingdom (Supplementary Table S7). This narrow geographical source of variety parents is consistent with the assertion of Fradgley *et al.* (2019) that breeders are using less variety parents from other countries, perhaps to increase the geographical and environmental adaptation of the UK gene pool.

Besides the limited geographical diversity of variety parents recently used in UK breeding, other studies have shown limited genetic diversity in important loci due to selection. For instance, Brinton *et al.* (2020) showed that UK RL varieties only contain three haplotype blocks in a chromosome 6A interval (187–445 Mbp) that is associated with productivity traits (*e.g.*, Simmonds *et al.* 2014). In contrast, the Watkin collection (landraces collected in the 1930s; Wingen *et al.* 2014) contains 40 haplotypes blocks, including Watkins-specific haplotypes with beneficial phenotypes that are currently not present in the worldwide germplasm evaluated in Brinton *et al.* (2020). Understanding the need to broaden the UK gene pool, UK breeders are actively pursuing the inclusion of new diversity from wheat landraces, synthetics, and wild relatives (Moore 2015). In addition to using these sources, it is important to note that increasing diversity can also be achieved by crossing with already adapted varieties carrying alien introgressions, like Robigus which carries an interspecific introgression fragment (Gardiner *et al.* 2019). Robigus has made significant contributions to the UK wheat pedigree since its introduction. Fradgley *et al.* (2019) identified Robigus as the second most used parents in UK breeding, next to Capelle Desprez.

We also observed a clear association between the population groups and end use groups. Varieties from the Claire and Robigus population groups are mostly used for biscuit (UFG3) and feed (UFG4). This is not surprising as Claire and Robigus themselves are biscuit-making (UFG3) varieties. Varieties from the Cadenza population group are mostly used for breadmaking (UFG1 and UFG2). This is consistent with the fact that Cadenza is a breadmaking variety. One probable explanation for this association is that breeders tend to make crosses with varieties from the same end-use groups to ensure that the gene combinations underlying the traits in the target end-use groups are preserved in their progenies (Simon Berry 2021, personal communication). This suggests that the choice of parents is an important determinant of the end-use class of varieties.

### Historical data could be valuable for trait mapping

We identified significant MTA peaks spanning a gene (*NAM-A1*) that have been previously associated with natural variation in time to senescence—synonymous to the days to ripening trait examined in our study. Cormier *et al.* (2015) identified a C/T missense SNP (in the NAC domain) and A/- frame-shift deletion (leading to a truncated protein) in *NAM-A1* from a worldwide wheat collection, and suggested functional roles for these polymorphisms. Harrington *et al.* (2019) showed that missense mutations in the NAC domain of *NAM-A1* result in delayed peduncle and flag leaf senescence. Similarly, Avni *et al.* (2014) showed that loss of function *NAM-A1* mutants showed significant delay in senescence. Given the large interval covered by the MTA peaks for days to ripening on chromosome 6A (73.4 – 86.5 Mbp, ∼140 genes), we cannot rule out the possibility that other gene(s) underly these days to ripening effect. Nonetheless, the co-localization of our GWAS peak with a known locus for the target trait highlights the usefulness of this historical dataset for quantitative trait mapping.

### Limitations of this study

Due to the type (gene-based SNP) and limited number of markers used, we acknowledge the limitation of this study to more precisely define the population groups represented in UK winter bread wheat collection to a high resolution. Brinton *et al.* (2020) demonstrated the inadequacy of array-based genotyping chips to precisely define haplotype groups due to their gene-centric design. Scaffold-level assemblies are now available for important UK wheat varieties including representatives of Pop1, Pop3, and Pop4 (Cadenza, Claire, and Robigus; Walkowiak *et al.* 2020). These genome assemblies can be combined with high-density genotyping or re-sequencing data to more precisely define the populations groups of wheat varieties grown in the United Kingdom.

Also, beside the MTA for days to ripening, we did not identify robust MTA for the other traits. This could be due to three main reasons. First, the sample size used in our study limits the statistical power of our GWAS analysis. As our study focused on a defined breeding period (2002 – 2017) our sample size was limited to the number of varieties trialed in this period. Using the functions described by Wang and Xu (2019) for explicitly calculating the power of a LMM-based GWAS analysis, we estimate that our population size can detect MTA that explains at least 14.9% of phenotypic variance at a statistical power of 0.9. Consistent with this, the most significant days to ripening MTA we detected explains 17.5% of variation in days to ripening. Our GWAS population size is therefore underpowered to detect QTL with smaller effects. Second, most such large effect genes controlling these traits will have been fixed in the United Kingdom wheat population over time by breeders. As such, the MTA detected here, although of large effect, may not have been under strong selection by breeders over time. Third, while the phenotyping conditions used in the NPVT might be representative of UK farming conditions, they might not always be best suited for trait mapping. An example is the application of PGRs in the trials to prevent lodging (by reducing plant height), but this might mask the effect of height genes. Despite these limitations, our work demonstrates that national trials data can be valuable for examining trait trends, stability, and genetic architecture.

## Data availability

The original data files for the trials described in this study can be downloaded from the AHDB website at: https://ahdb.org.uk/knowledge-library/recommended-lists-for-cereals-and-oilseeds-rl-harvest-results-archive (Accessed: 2021 December 13). As data for different traits are combined in these original files, we reorganized the files to separate the data for each trait into separate files. The reorganized files are available at Zenodo: https://doi.org/10.5281/zenodo.4761528 (Accessed: 2021 December 13). All Supplementary figures and tables cited, as well as the pedigree file for Helium visualization, are provided as Supplementary Files available with this article. Supplementary material is available at figshare: https://doi.org/10.25387/g3.14946033 (Accessed: 2021 December 13).
